# Accumulation of the PX domain mutant Frank-ter Haar syndrome protein Tks4 in aggresomes

**DOI:** 10.1186/s12964-015-0108-8

**Published:** 2015-07-17

**Authors:** Csaba Ádám, Anna Fekete, Gábor Bőgel, Zsuzsanna Németh, Natália Tőkési, Judit Ovádi, Károly Liliom, Szabolcs Pesti, Miklós Geiszt, László Buday

**Affiliations:** Institute of Enzymology, Research Centre for Natural Sciences, Hungarian Academy of Sciences, Magyar tudósok körútja 2, Budapest, 1117 Hungary; Department of Medical Chemistry, Semmelweis University Medical School, Budapest, Hungary; Department of Physiology, Semmelweis University Medical School, Budapest, Hungary; “Lendület” Peroxidase Enzyme Research Group of the Semmelweis University and the Hungarian Academy of Sciences, Budapest, Hungary

**Keywords:** Tks4, Aggresome, Frank-ter Haar Syndrome, Microtubules, Misfolded protein

## Abstract

**Background:**

Cells deploy quality control mechanisms to remove damaged or misfolded proteins. Recently, we have reported that a mutation (R43W) in the Frank-ter Haar syndrome protein Tks4 resulted in aberrant intracellular localization.

**Results:**

Here we demonstrate that the accumulation of Tks4^R43W^ depends on the intact microtubule network. Detergent-insoluble Tks4 mutant colocalizes with the centrosome and its aggregate is encaged by the intermediate filament protein vimentin. Both the microtubule inhibitor nocodazole and the histone deacetylase inhibitor Trichostatin A inhibit markedly the aggresome formation in cells expressing Tks4^R43W^. Finally, pretreatment of cells with the proteasome inhibitor MG132 markedly increases the level of aggresomes formed by Tks4^R43W^. Furthermore, two additional mutant Tks4 proteins (Tks4^1–48^ or Tks4^1–341^) have been investigated. Whereas the shorter Tks4 mutant, Tks4^1–48^, shows no expression at all, the longer Tks4 truncation mutant accumulates in the nuclei of the cells.

**Conclusions:**

Our results suggest that misfolded Frank-ter Haar syndrome protein Tks4^R43W^ is transported via the microtubule system to the aggresomes. Lack of expression of Tks4^1–48^ or aberrant intracellular expressions of Tks4^R43W^ and Tks4^1–341^ strongly suggest that these mutations result in dysfunctional proteins which are not capable of operating properly, leading to the development of FTHS.

## Background

Cells deploy quality control mechanisms to remove damaged or misfolded proteins. These mechanisms include up-regulated chaperones to facilitate protein refolding, ubiquitin-dependent degradation of misfolded/damaged proteins, and formation of detergent insoluble aggresomes at the juxtanuclear region [1–4]. Aggresome formation is a vital mechanism to eliminate misfolded proteins, nevertheless, it is not clear if it is an independent process or it is activated only when the degradative capacity of the ubiquitin-proteasome system is overwhelmed [5]. In this system, misfolded and aggregated proteins are selectively recognized, ubiquitinated, and delivered via HDAC6 (histone deacetylase 6)-dependent, microtubule-based transport toward the microtubule-organizing center (MTOC) [6]. Aggresome formation not only protects cells from proteotoxicity but also facilitates the clearance of damaged proteins by autophagy [6]. The accumulation of protein aggregates is commonly linked with various human diseases referred to as protein conformation disorders [7].

Frank-ter Haar syndrome (FTHS) is a rare disorder associated with cardiovascular, skeletal and craniofacial anomalies including macrocornea with or without glaucoma, brachycephaly, large anterior fontanels, hypertelorism, anteverted nostrils, thoracolumbar kyphosis, and short hands [8, 9]. Protruding simple ears and prominent coccyx bone can be also regarded as important diagnostic signs [8, 9]. FTHS patients unfortunately die in infancy or in early childhood due to cardiovascular anomalies or respiratory infections. The most common underlying genetic defect in FTHS has recently been established through homozygosity mapping studies in patients, identifying homozygous mutations in the *SH3PXD2B* gene on chromosome 5q35.1 [9]. The analysis of patients identified four different intragenic mutations, and one complete deletion of *SH3PXD2B* [9]. A novel mutation in FTHS patients has also been described caused by the deletion of exon 13 of the SH3PXD2B gene [10]. Recently, two new homozygous loss-of-function mutations have been identified in the SH3PXD2B gene in patients with Borrone dermato-cardio-skeletal syndrome (BDSC syndrome) which is related to the FTHS [11]. *SH3PXD2B* null mice appear to share many of the skeletal, craniofacial, cardiac and ocular defects described in FTHS, supporting the link between this gene and the syndrome [9]. Interestingly, studying the spontaneous mouse mutant *nee* has revealed that those mice also exhibited runted growth, craniofacial and skeletal abnormalities, ocular anterior segment dysgenesis, and hearing impairment, similar to *SH3PXD2B* null mice [12]. Using genetic mapping and DNA sequencing, the cause of *nee* phenotypes was identified as a 1 bp deletion within the *SH3PXD2B* gene on mouse Chromosome 11 which causes a frameshift and a protein truncation altering a portion of the third SH3 domain and deleting all of the fourth SH3 domain [12].

The protein product of the *SH3PXD2B* gene is known as Tks4/HOFI/SH3PXD2B/fad49 (tyrosine kinase substrate with four SH3 domains/homolog of FISH/SH3 and PX domain-containing protein 2B/factor for adipocyte differentiation 49, hereafter termed Tks4). Tks4 has emerged as a candidate scaffold molecule that has the capability to regulate the actin cytoskeleton via Src and EGFR [13–15]. In addition, Tks4 was shown to play an important role in the formation of functional podosomes [16], production of reactive oxygen species (ROS) by tumor cells [17–19], and in the differentiation of white adipose tissue [20]. In 2010, Iqbal and colleagues have identified a family with FTHS whose *SH3PXD2B* gene contains a substitution mutation which results in the change of the conserved arginine 43 to tryptophan in the PX domain [9]. We have recently demonstrated that the R43W mutation seriously impairs the cellular expression and the function of Tks4 [14].

In the present study we further characterized the mutant Tks4^R43W^ protein in COS7 cells. Here we show that mutant Tks4 is very likely misfolded since it is seen in the detergent-insoluble fraction of cell extracts. In addition, in some cells Tks4^R43W^ colocalizes with microtubules and the perinuclear Tks4 aggregate formation depends on the intact microtubule network. The misfolded Tks4 mutant also colocalizes with the MTOC and its aggregate is encaged by the intermediate filament protein vimentin. Finally, pretreatment of cells with the proteasome inhibitor MG132 markedly increases the levels of aggresomes formed by Tks4^R43W^. Our results therefore suggest that the misfolded FTHS protein Tks4^R43W^ is transported via the microtubular system to aggresomes localized at the juxtanuclear region.

## Results

### Tks4^R43W^ displays characteristics of misfolding

**Fig. 1:**
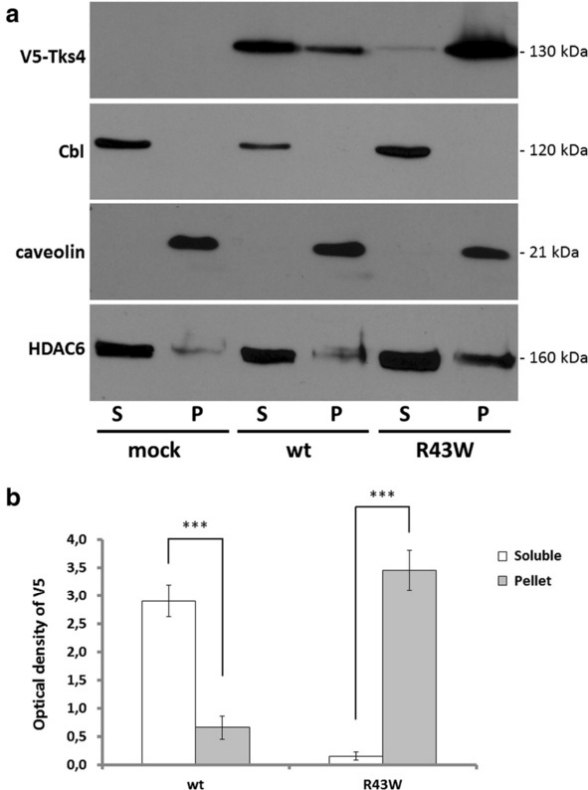
The Tks4^R43W^ mutant protein is present in detergent-insoluble pellets. **1a**, COS7 cells were transiently transfected with wild type V5-Tks4 or V5-Tks4^R43W^ constructs. After 18 h the cells were lysed, and the lysates were separated into detergent-soluble (S) and insoluble pellet fractions (P) by centrifugation and analyzed by Western blotting for Tks4 (with anti-V5 antibody), Cbl, caveolin-1 and HDAC6, respectively. **1b**, Wild type and mutant Tks4 protein levels were determined by densitometry. Error bars represent the standard deviation from three independent experiments. Asterisks indicate the level of significance (one asterisk, *P* < 0.05; two asterisks, *P* < 0.01; three asterisks, *P* < 0.001)

In 2010, Iqbal and colleagues have identified a patient with FTHS whose *SH3PXD2B* gene contains a substitution mutation which results in the change of the conserved arginine 43 to tryptophan in the PX domain. Interestingly, the symptoms of this particular patient were indistinguishable of those who had more severe mutations leading to complete loss of Tks4 protein synthesis [9]. This finding suggests that the Tks4^R43W^ protein is likely damaged or misfolded, leading to reduced intracellular protein concentration. We have previously expressed Tks4^R43W^ in COS7 cells and found that the expression of the mutant protein was significantly decreased in cell lysates, it formed aggregates and accumulated around the nucleus [14]. In this study Tks4^R43W^ protein was further characterized concerning its intracellular behavior. Damaged or misfolded proteins in aggresomes are often insoluble in non-denaturing detergents [4]. Therefore, we investigated the solubility of V5-tagged mutant Tks4 by sequential extraction into detergent-soluble and insoluble fractions. As shown in Fig. 1, wild type Tks4 was found predominantly in the detergent soluble fraction. However, Tks4^R43W^ was sedimented almost exclusively in the detergent-insoluble pellet fraction. To exclude experimental artifact, the solubility of a well-characterized cytosolic protein was also determined. The E3 ubiquitin-protein ligase Cbl was detected completely in the soluble fractions (Fig. 1, [21, 22]). In addition, a marker of the pellet fraction (caveolin) and an aggresome marker (HDAC6) were also tested.

### Different localization patterns of Tks4^R43W^ protein in COS7 cells

**Fig. 2:**
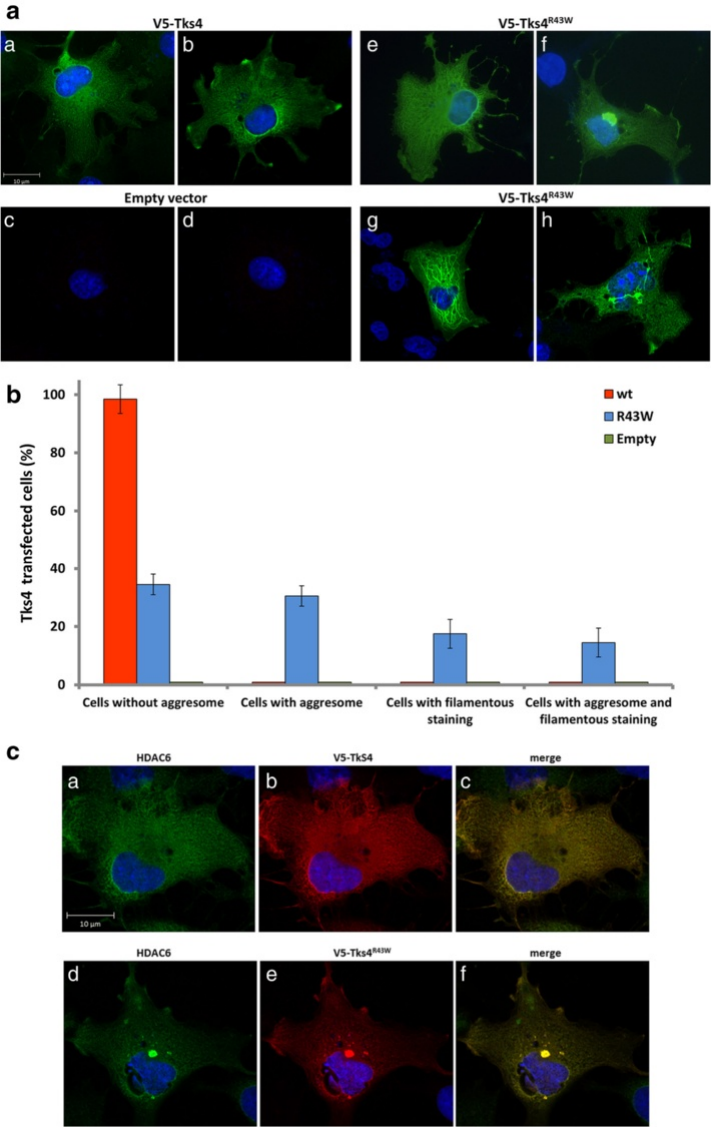
Different localization patterns of Tks4 and Tks4^R43W^ proteins in cells. **2a**, COS7 cells were transiently transfected with V5-Tks4, V5-Tks4^R43W^ or V5-empty constructs. After 18 h, cells were fixed and stained for Tks4 with anti-V5 antibody. **2b**, The percentage of aggresomes in cells was quantified by counting 300 cells/sample by using ImageJ software. Error bars represent the standard deviation from three independent experiments. **2c**, Tks4^R43W^ aggresomes colocalize with the HDAC6 aggresome marker. COS7 cells were transiently transfected with V5-Tks4 or V5-Tks4^R43W^ constructs. After 18 h, cells were fixed and stained stained for Tks4 (with anti-V5 antibody, *red*) and HDAC6 (*green*)

We have recently demonstrated that the R43W mutant Tks4 aggregates in cells and the aggregate localizes at the juxtanuclear region [14]. However, only a part of Tks4^R43W^-expressing cells showed this localization pattern. Therefore, we transiently expressed the V5-tagged mutant Tks4 in cells, stained them with anti-V5 antibody, and categorized them based on the localization patterns of Tks4^R43W^. We can distinguish the following localization patterns: a, cells with uniform cytoplasmic distribution (Fig. 2a/*e*), b, cells with aggresomes (Fig. 2a/*f*), c, cells with filamentous staining (Fig. 2a/*g*), finally, d, cells with aggresomes and filamentous anti-V5 staining together (Fig. 2a/*h*). To calculate the percentage of cells showing different Tks4^R43W^ localization, cells with V5-Tks4 immunreactivity were scrutinized under laser confocal microscope. We found that approximately 35 % of cells showed uniform cytoplasmic distribution, while the percentage of cells containing aggressomes was around 32 %. The percentage of cells with filamentous staining only or with aggresomes was significantly lower, approximately 18 and 15 %, respectively (Fig. 2b). Taken together, it seems that approximately half of the cells expressing Tks4^R43W^ contain aggresomes. To ensure that we can detect Tks4^R43W^ in the aggresomes, HDAC6, a marker of aggresomes, was also stained (Fig. 2c).

### The microtubule network delivers Tks4 ^R43W^ to aggresomes

**Fig. 3:**
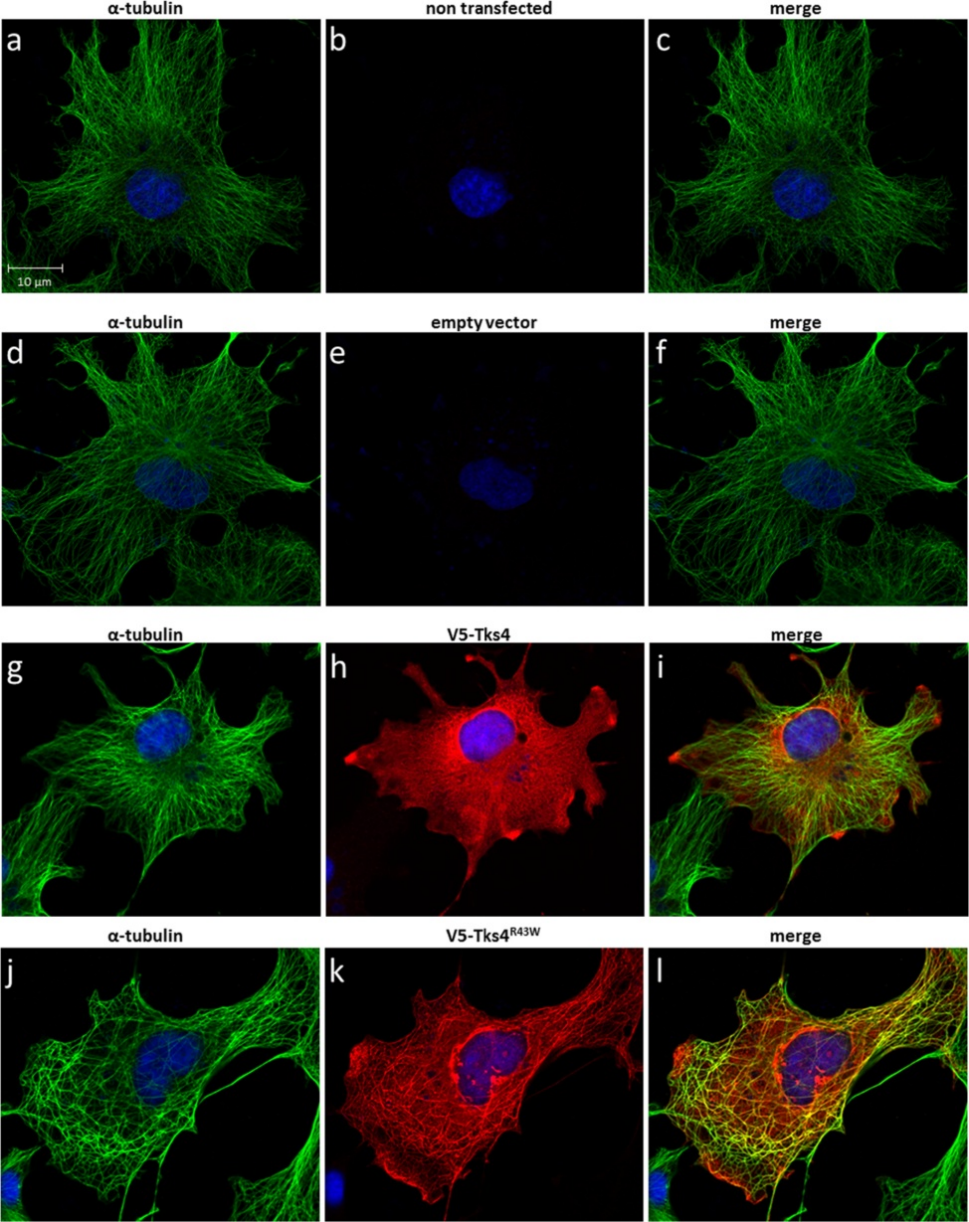
The Tks4^R43W^ mutant protein colocalizes with the microtubule network of the cytoskeleton. COS7 cells were transiently transfected with wild type V5-Tks4 (**3g**, **h**, **i**), V5-Tks4^R43W^ (**3j**, **k**, **l**) or V5-empty (**3d**, **e**, **f**) constructs. Non-transfected cells are also shown (**3a**, **b**,**c**). After 18 h, cells were fixed and stained for Tks4 (with anti-V5 antibody, red, **3b**, **e**, **j**, **k**) and α-tubulin (green, **3a**, **d**, **g**, **j**). Merged pictures are also shown (**3c**, **f**, **i**, **l**). Cell nuclei were visualized by DAPI staining. The scale bar represents 10 μm

Damaged or misfolded proteins are delivered via an HDAC6-dependent, microtubule-based retrograde transport toward the microtubule-organizing center (MTOC) into aggresomes [6]. Using anti-alfa-tubulin fluorescent staining we have shown that, in those cells where the mutant Tks4 shows filamentous staining, Tks4^R43W^ partially colocalizes with the microtubule network, while the wild type protein shows a uniform cytoplasmic distribution (Fig. 3).

**Fig. 4:**
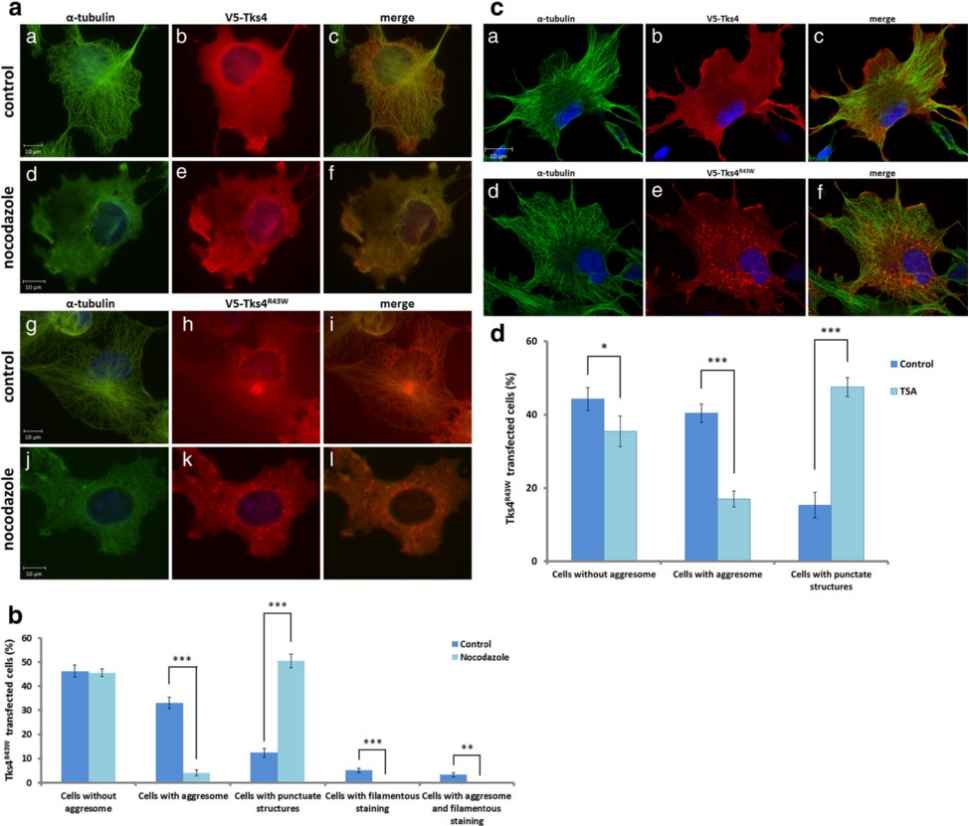
Perinuclear Tks4^R43W^ aggregate formation is microtubule and HDAC6-dependent. **4a**, COS7 cells were transiently transfected with wild type V5-Tks4 or V5-Tks4^R43W^ constructs. After 4 h, the cells were treated with 1,6 μM nocodazole for 16 h. The cells were fixed and stained for Tks4 (with anti-V5 antibody, red) and α-tubulin (*green*). **4b**, The percentage of aggresomes and punctate structures in cells was quantified by counting 300 cells/sample by using ImageJ software. **4c**, COS7 cells were transiently transfected with wild type V5-Tks4 or V5-Tks4^R43W^ constructs. After 4 h, the cells were treated with 1 μM Trichostatin A (TSA) for 16 h. The cells were fixed and stained for Tks4 (with anti-V5 antibody, red) and α-tubulin (*green*). **4d**, The percentage of aggresomes and punctate structures in cells was quantified by counting 300 cells/sample by using ImageJ software. Error bars represent the standard deviation from three independent experiments. Asterisks indicate the level of significance (*one asterisk*, *P* < 0.05; *two asterisks*, *P* < 0.01; *three asterisks*, *P* < 0.001)

In order to show that the transport of Tks4^R43W^ is indeed microtubule dependent, we pretreated COS7 cells with 1.6 μM of the microtubule depolymerizing drug nocodazole for 16 h, which leads to destabilization of the microtubule network (Fig. 4a). In the presence of nocodazole, aggresome formation in Tks4^R43W^-expressing cells was largely inhibited (Fig. 4a/*j*-*l*, 4b). Interestingly, in those cells protein aggregates of the mutant Tks4 showed punctate structures throughout the cell, reflecting that without active microtubule network small aggregates of Tks4^R43W^ cannot be delivered to the MTOC and assembled to large cytoplasmic inclusions. To prove the role of microtubules in the transport of Tks4^R43W^ in another way, cells expressing Tks4^R43W^ were treated with the histone deacetylase inhibitor Trichostatin A (TSA). TSA is a well-known inhibitor of HDAC6 that yields hyper-acetylated tubulin. It was found that hyper-acetylated microtubules observed after TSA treatment exhibited delayed depolymerization [23–25]. Figure 4c and Fig. 4d demonstrate that inhibition of HDAC6 in Tks4^R43W^–expressing cells prevented aggresome formation. Interestingly, misfolded Tks4 protein shows very similar staining in these cells as seen in cells treated with the microtubule depolymerizing drug nocodazole.

### The aggresome formed by Tks4^R43W^ colocalizes with the centrosome

**Fig. 5:**
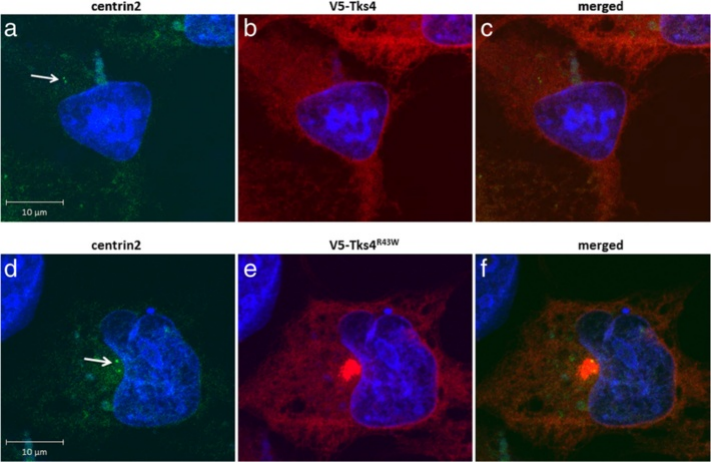
Aggresomes formed by Tks4^R43W^ mutant colocalizes with the centrosome. COS7 cells were transiently transfected with hCent2-pEGFP-C1 and V5-Tks4 (**5a**, **b**, **c**) or hCent2-pEGFP-C1 and V5-Tks4R43W (**5d**, **e**, **f**) constructs. After 18 h, cells were fixed and stained for Tks4 with anti-V5 antibody (*red*). Cell nuclei were visualized by DAPI staining. The arrow indicates the localization of centrin2 present at the juxtanuclear region. The scale bar represents 10 μm

As described above, aggresomes are typically formed at the MTOC at the juxtanuclear region. MTOC or centrosome contains several specific proteins responsible for microtubule nucleation and anchoring, including γ-tubulin, pericentrin, ninein and centrins [26]. To prove that Tks4^R43W^ aggregates are accumulated at the MTOC, the centrosome marker protein centrin2 was fused to GFP and was co-expressed with the wild type Tks4 or Tks4^R43W^ in COS7 cells. As demonstrated in Fig. 5, aggresome formed by mutant Tks4 showed clear colocalization with centrosome marker protein centrin2.

### The Tks4^R43W^ aggresome is encaged by vimentin

**Fig. 6:**
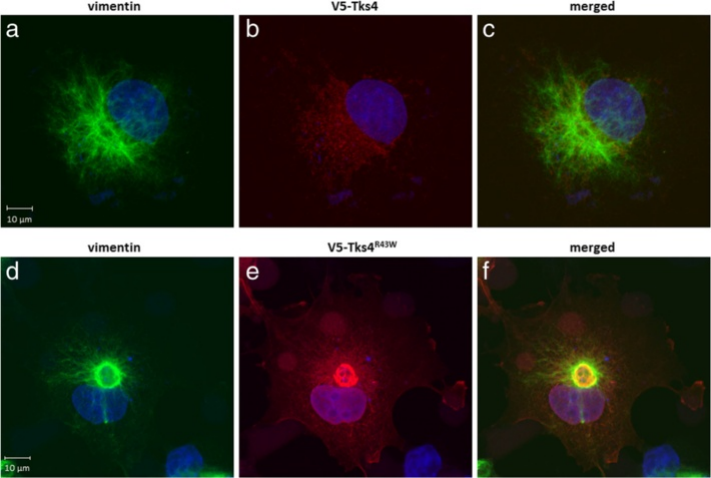
Tks4^R43W^ aggresome is encaged by vimentin. COS7 cells were transiently transfected with wild type V5-Tks4 (**6a**, **b**, **c**) or V5-Tks4^R43W^ (**6d**, **e**, **f**) constructs. After 18 h, cells were fixed and stained for Tks4 (with anti-V5 antibody, *red*) and vimentin (*green*). Cell nuclei were visualized by DAPI staining. The scale bar represents 10 μm

One of the most characteristic features of aggresomes is the localization of the intermediate filament protein vimentin which changes its normal fibrillar distribution to form a cage surrounding the aggregates [3, 4]. To further verify that the observed pericentriolar Tks4^R43W^ aggregate corresponds to aggresomes, subcellular localization of vimentin was examined in cell expressing wild type and mutant Tks4. In cells expressing wild type Tks4, vimentin distribution was filamentous and dispersed throughout the cell (Fig. 6a and c). However, in cells transfected with TKS4^R43W^ vimentin filaments were seen to form a ring-like structure around the aggregated proteins in the juxtanuclear region (Fig. 6d and f).

### The proteasome inhibitor MG132 increases the level of aggresome formation by Tks4^R43W^

**Fig. 7:**
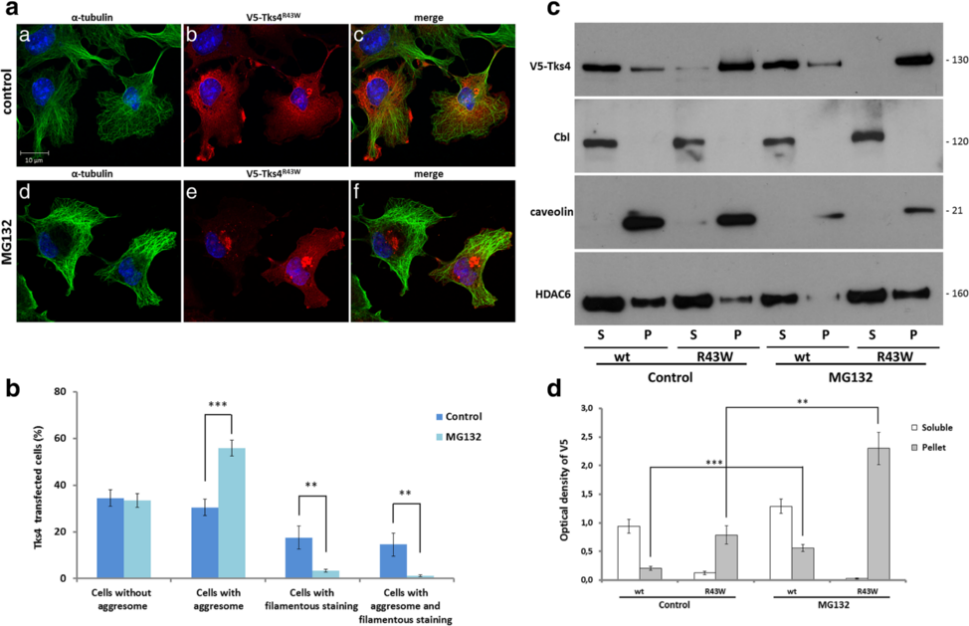
The proteasome inhibitor MG132 increases the level of aggresome formation by the Tks4^R43W^ mutant. **7a**, COS7 cells were transiently transfected with a V5-Tks4 and V5-Tks4^R43W^ construct. After 18 h, the cells were treated with 5 μM MG132 for 20 h. The cells were fixed and stained for Tks4 (with anti-V5 antibody, *red*) and α-tubulin (*green*). Cell nuclei were visualized by DAPI staining. **7b**, The percentage of cells with aggresomes was quantified by counting 300 cells/sample by using the ImageJ software. **7c**, COS7 cells were transiently transfected with V5-Tks4 and V5-Tks4^R43W^ construct. After 18 h the cells were treated with 5 μM MG132 for 20 h or left untreated. Cells were then lysed, and the lysates were separated into detergent-soluble (S) and insoluble pellet fractions (P) by centrifugation and analyzed by Western blotting for Tks4 (with anti-V5 antibody), Cbl, caveolin-1 and HDAC6, respectively. **7d**, The Tks4 protein levels were determined by densitometry. Error bars represent the standard deviation from three independent experiments. Asterisks indicate the level of significance (*one asterisk*, *P* < 0.05; *two asterisks*, *P* < 0.01; *three asterisks*, *P* < 0.001)

In our previous experiments we have shown that Tks4^R43W^ is transported to the centrosome where it can be sequestered in aggresomes. Nevertheless, other mechanisms, such as proteasomal degradation may also contribute to the clearance of this misfolded protein. To test if the proteasomal system significantly contributes to the elimination of Tks4^R43W^, we challenged the cells with the cell-permeable proteasome inhibitor MG132. Figure 7a and b show that pretreatment of the cells with 5 μM MG132 for 20 h significantly increased the percentage of cells with perinuclear aggregates. To see the effect of MG132 on protein levels too, cells treated with the inhibitor were separated into detergent soluble and insoluble fractions, as performed in Fig. 1. Figure 7c and d demonstrates that the protein levels present in the pellet fractions of MG132-treated cells were significantly increased in cells expressing the mutant Tks4 proteins. These findings suggest that the ubiquitin-proteasome system also contributes to the elimination of the mutant Tks4 proteins.

### Other mutations of the FTHS patients also result in aberrant expression of Tks4 proteins

**Fig. 8:**
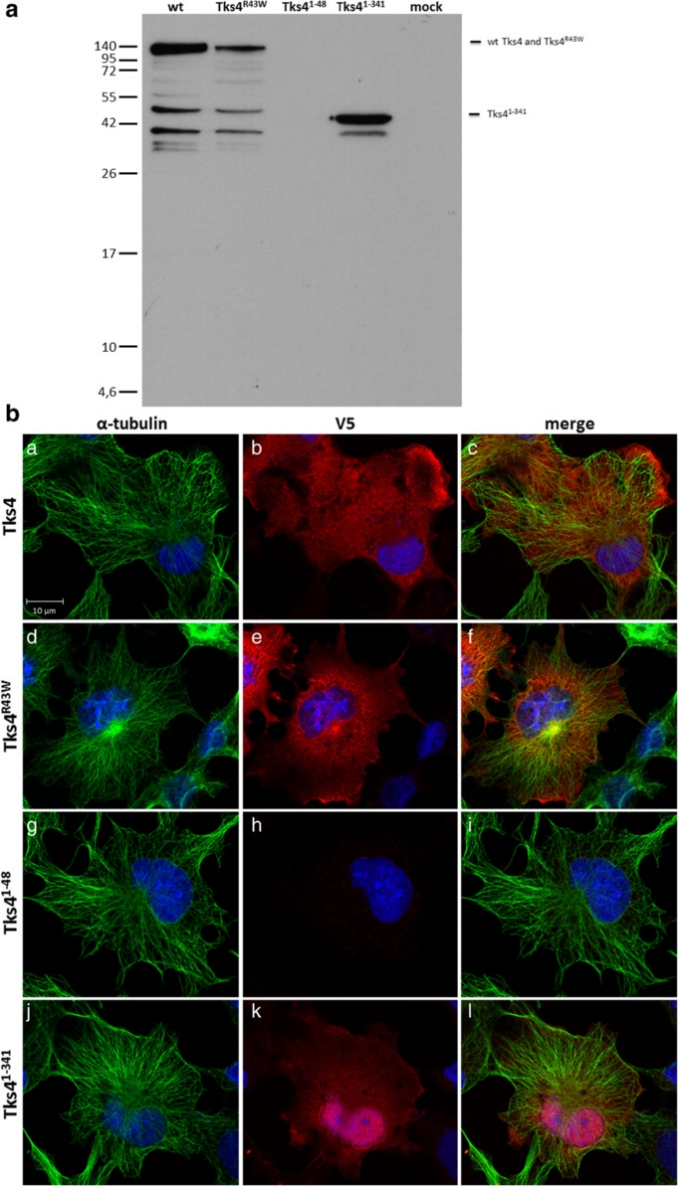
The expression patterns of Tks4 mutant proteins. **8a**, COS7 cells were transiently transfected with V5-Tks4, V5-Tks4^R43W^, V5-Tks4^1–48^ and V5-Tks4^1–341^ constructs. After 18 h the cells were lysed, the lysates were separated by centrifugation and the supernatant analyzed by Western blotting for Tks4 with anti-V5 antibody. **8b**, COS7 cells were transiently transfected with V5-Tks4, V5-Tks4^R43W^, V5-Tks4^1–48^ and V5-Tks4^1–341^ constructs. After 18 h, cells were fixed and stained for Tks4 (with anti-V5 antibody, *red*) and α-tubulin (*green*). Cell nuclei were visualized by DAPI staining. The scale bar represents 10 μm

In addition to the R43W mutation, *Iqbal* et al. described two further mutations in the *SH3PXD2B* gene of FTHS patients. A homozygous insertion c.147insT was detected which predicted the creation of an immediate stop in the same codon resulting a 48 amino acid long protein (Tks4^1–48^). In other patients, a homozygous 1 bp deletion c.969delG was identified which predicted a frameshift followed by a premature stop codon (p.G323fsX19). The expected protein possesses 341 amino acids (Tks4^1–341^) and the truncation occurs after the second SH3 domain in the structure of Tks4 [9]. To check the expression and the intracellular localization of these mutants, cDNA constructs were generated as described in the Methods. V5- Tks4^1–48^ and V5- Tks4^1–341^ were transiently expressed in COS7 cells, protein lysates were then subjected to SDS-PAGE and immunoblot with an anti-V5 monoclonal antibody. As demonstrated in Fig. 8a, wild type Tks4 and Tks4^R43W^ can be detected with an apparent molecular weight of 140 kDa. It is worth to note that due to the over-expression of the proteins some degradation can be seen at lower molecular weights. Interestingly, the shorter Tks4 mutant, Tks4^1–48^, shows no expression at all. Finally, expression of the Tks4^1–341^ can be detected with an approximate molecular weight of 45 kDa. Next, we transiently expressed the V5-tagged Tks4 proteins in cells. As Fig. 8b demonstrates, a portion of Tks4^R43W^ localizes at the juxtanuclear region in the aggresome, as described above (Fig. 2a). Tks4^1–48^, as expected, did not express in the cell. Finally, Tks4^1–341^ shows an interesting picture accumulating in the nuclei of the cells. Taken together, while Tks4^1–48^ shows no expression in the cells, the other two mutants, Tks4^R43W^ and Tks4^1–341^, show abnormal intracellular localizations.

## Discussion

FTHS is an autosomal-recessive disorder characterized by abnormalities that affect bone, heart, and eye development. Patients usually die in infancy or in early childhood because of the cardiovascular anomalies, respiratory infections or unknown causes [9]. Iqbal and colleagues have investigated several families with FTHS and revealed five different homozygous mutations in the *SH3PXD2B* gene encoding for Tks4 [9]. One particularly interesting mutation of them is the change of the highly conserved arginine 43 to tryptophan within the PX domain of Tks4 [9]. We have recently demonstrated that the R43W mutation seriously impairs the lipid-binding of the PX domain and the cellular expression of Tks4 [14].

In the present study we have further characterized the mutant Tks4^R43W^ protein and studied the mechanism by which the misfolded Tks4 is sequestered. We prove here that the Tks4^R43W^ protein is transported via the microtubule network to aggresomes. This is based on the following findings: a, Tks4^R43W^ shows partial colocalization with the microtubule network; b, both nocodazole, a microtubule-depolymerizing drug, and Trichostatin A, a histone deacetylase inhibitor, inhibit aggresome formation in cells expressing the mutant Tks4; c, Tks4^R43W^ shows colocalization with the centrosome-specific centrin2; d, finally, the intermediate filament protein vimentin forms a ring-like structure around the Tks4^R43W^ aggregates.

Much evidence has accumulated to indicate that the aggresome represents a special protective response to proteotoxic stresses [1, 2, 4, 27]. However, it is not clear if aggresome formation is an independent process or it is activated only when the degradative capacity of the ubiquitin-proteasome system is overwhelmed. Our data based on the characteristics of the Tks4^R43W^ favor the second alternative. Pretreatment of cells with the cell-permeable proteasome inhibitor MG132 resulted in the dramatic increase in the number of Tks4^R43W^ expressing cells with perinuclear aggregates. Therefore, it is likely that mutant Tks4 is also eliminated by the ubiquitin-proteasome system. However, immunoblotting of the immunopericipated Tks4^R43W^ with specific anti-ubiquitin antibody did not detect the ubiquitination of the mutant Tks4 (data not shown). Further experiment will therefore be required to establish the precise mechanism by which the proteasome system eliminates the misfolded Tks4.

Finally, we have constructed two other mutant Tks4 proteins which were predicted earlier by studying FTHS patients [9]. We wished to investigate how they express in cells and what their intracellular localization is. The V5-tagged, 48 amino acid long protein (Tks4^1–48^) showed no expression at all in cells studied either by immunoblot or by confocal microscopy. However, when the V5-tagged, 341 amino acid long truncation mutant (Tks4^1–341^) were transiently expressed in COS7 cell, and cell lysates were prepared and subjected to anti-V5 immunoblot, a specific band was detected with an approximate molecular weight of 45 kDa. Interestingly, Tks4^1–341^ was localized primarily in the nuclei of the cells. The expression levels of the shorter truncation mutant Tks4 (c.147insT, Tks4^1–48^) were analyzed earlier by immunoblot of total cell lysates of human primary dermal fibroblasts isolated from FTHS patient. While Tks4 signal was detected in control fibroblasts, no signal was seen in cells of FTHS patients [9]. Our finding using transiently over-expressed cell system is in agreement with the above finding, namely that we could also not detected the expression of Tks4^1–48^ protein in cells. Interestingly, *Iqbal* et al. detected normal levels of *SH3PXD2B* transcript in FTHS families with the c.147insT (F49X) mutation, excluding that the premature stop codon introduced by this mutation results in nonsense-mediated RNA decay. They guessed that the truncation resulted in an unstable protein [9]. Taken together, lack of expression of Tks4^1–48^ or aberrant intracellular expressions of Tks4^R43W^ and Tks4^1–341^ strongly suggest that these mutations result in dysfunctional proteins which are not capable of operating properly, leading to the development of FTHS.

It has been well established that both Tks4 and Tks5 play important roles in the regulation and formation of podosomes [16, 28, 29]. Podosomes represent transient subcellular structures that are actin-rich membrane protrusions found on the ventral surface of the cells facing the extracellular matrix. The migration of normal cells is driven by podosomes when these specialized structures allow the cells to adhere and invade in the surroundings [29]. Since podosomes are necessary for the adhesion and migration of a variety of cell types, including macrophages, dendritic cells, osteoclasts, vascular smooth muscle, and endothelial cells [9], it is likely that dysfunctional Tks4 proteins are implicated in the development of FTHS phenotype.

## Conclusions

In summary, we show that the mutant FTHS protein Tks4^R43W^ is found in the detergent-insoluble cell fractions. The misfolded protein seems to be transported to the aggresomes via the microtubule system. Lack of expression of Tks4^1–48^ or aberrant intracellular expressions of Tks4^R43W^ and Tks4^1–341^ strongly suggest that these mutations result in dysfunctional proteins which are not capable of operating properly, leading to the development of FTHS.

## Methods

### Antibodies, constructs and reagents

Monoclonal and polyclonal antibodies against the V5 epitope (R96025 and AB3792) were purchased from Invitrogen (Carlsbad, CA, USA) and Millipore (Billerica, MA, USA), respectively. Antibody against α-tubulin (DM1A) and vimentin (V6630) were obtained from Sigma-Aldrich (St. Louis, MO, USA). Antibody against Cbl (sc-170) was from Santa Cruz Biotechnology, Inc. (Santa Cruz, CA, USA). Antibody against HDAC6 (7558) and caveolin-1 (3267) were obtained from Cell Signaling Technology (Danvers, MA, USA). The anti-rabbit and anti-mouse HRP-linked antibodies (NA934V, NA931V) were purchased from GE Healthcare (Little Chalfont, Buckinghamshire, United Kingdom). Alexa Fluor 488 anti-rabbit (A11034), Alexa Fluor 488 anti-mouse (A11029), Alexa Fluor 546 anti-rabbit (A11035), and Alexa Fluor 546 anti-mouse (A11030) antibodies were purchased from Invitrogen (Carlsbad, CA, USA). The generation of V5 epitope tagged Tks4 and the V5-Tks4^R43W^ mutant was described previously [13, 14]. To generate the V5-tagged Tks4^1–48^ and Tks4^1–341^ proteins [9], the shorter Tks4 coding sequences was amplified by PCR and subcloned into BamHI/XbaI sites of pcDNA3.1/TOPO-V5-His plasmid (Life Technologies, Carlsbad, CA, USA). The single guanine change was introduced into the c.969delG deletion mutant-V5 construct (V5- Tks4^1–341^) using the QuikChange Lightning Site-Directed Mutagenesis Kit (Agilent Technologies). The constructs were verified by sequencing. The human centrin2 containing hCent2-pEGFP-C1 construct was a kind gift of Jeffrey L. Salisbury (Tulane University, USA). Stock solutions of MG132 (474790, Calbiochem), nocodazole (M1404, Sigma-Aldrich) and Trichostatin A (T8552, Sigma-Aldrich) were prepared according to the manufacturer’s instructions. Pfu DNA polymerase (EP0501) was purchased from Thermo Scientific (Waltham, MA, USA). The BamHI (1010A) and XbaI (1093A) enzymes were purchased from Takara Bio Inc (Otsu, Shiga, Japan).

### Cell lines, transfection, and inhibition

COS7 cells were purchased from American Type Culture Collection and maintained in Dulbecco’s modified Eagle’s medium (DMEM) supplemented with 10 % fetal calf serum (Invitrogen, Carlsbad, CA, USA), penicillin (100 units/ml), and streptomycin (100 μg/ml). All cell lines were transiently transfected with Lipofectamine (Invitrogen) according to the manufacturer’s instructions. In the experiments where indicated, transfected cells were pretreated with 5 μM MG132 for 20 h, 1.6 μM nocodazole for 16 h or 1 μM Trichostatin A for 16 h after the transfection.

### Confocal microscopy

COS7 cells plated on glass cover slips were transiently transfected with different constructs as indicated and then incubated for 4 or 18 h. When indicated 5 μM MG132 were added 18 h after transfection for 20 h. In other experiments cells were treated with 1.6 μM nocodazole or with 1 μM Trichostatin A for 16 h starting 4 h after transfection. The cells were fixed in 4 % paraformaldehyde-PBS for 15 min or in −20 °C methanol for 5 min (in centrosome colocalization experiment), permeabilized in 0.2 % Triton X-100 in PBS for 5 min, and blocked with 1 % BSA in PBS for 20 min. Anti-V5 polyclonal rabbit and anti-α-tubulin antibody was applied in 1:1000 dilution for 30 min. After washing with PBS the samples were incubated with Alexa Fluor 488 (or 546) labeled anti-mouse or anti-rabbit secondary antibody for 30 min in 1:1000 dilution. After 30 min of washing with PBS cover slips were mounted onto slides in a 100 mM Tris–HCl buffer, pH 8.5, containing 10 % Mowiol 4–88 (Calbiochem), 25 % glycerol, and 2.5 % 1,4-diazobicyclo- [2.2.2]octane (DABCO, Sigma-Aldrich, St. Louis, MO, USA). The pictures of fixed samples were acquired on a Zeiss LSM710 inverted confocal microscope with 63X objective (Carl Zeiss, Jena, Germany). To minimize the cross-talk between imaged channels, sequential image collection was used. Cells are shown as single confocal section. All images were processed using ZEN software (Carl Zeiss, Jena, Germany).

### Characterization of aggregates and western blotting

The sequential extraction of aggregates was performed according to [30]. Equal volumes of protein extracts from each fraction (supernatant, pellet) were subjected to SDS-PAGE and transferred to nitrocellulose membranes (Bio-Rad, Hercules, CA, USA). Membranes were then blocked with 5 % skim milk powder, incubated with monoclonal anti-V5, Cbl, caveolin-1 or HDAC6 primary antibodies, and horseradish peroxidase-conjugated secondary antibodies, and developed using ECL detection reagents (GE Healthcare, Little Chalfont, Buckinghamshire, United Kingdom). Densitometry on scanned images was done using the ImageJ 1.46x software.

### Statistics

The number of aggresomes was quantified by counting 300 cells/sample by using ImageJ software. Quantitative results are presented as mean and s.d. of at least three independent experiments. Statistical differences between two groups of data were analyzed by Student’s *t*-test.

## Abbreviations

Tks4: Tyrosine kinase substrate with four SH3 domains
HDAC6: Histone deacetylase 6
MTOC: Microtubule-organizing center
TSA: Trichostatin A
GFP: Green fluorescent protein
PX domain: Phox homology domain
SH3 domain: Src homology domain
